# Mouse-tracking as a tool for investigating strategic behavior in Public Goods Game: an experimental pilot study

**DOI:** 10.3389/fpsyg.2025.1635677

**Published:** 2025-09-22

**Authors:** Asma Benachour, Vladimir Medvedev, Oksana Zinchenko

**Affiliations:** 1 Laboratory of Brain-Computer Interfaces, Skolkovo Institute of Science and Technology, Moscow, Russia; 2 Centre for Neurocognitive Research, Moscow State University of Psychology & Education (MSUPE), Moscow, Russia; 3 Centre for Cognition and Decision Making, Institute for Cognitive Neuroscience, HSE University, Moscow, Russia

**Keywords:** mouse-tracking, Public Goods Game, decision-making, cooperation, strategic behavior

## Abstract

**Introduction:**

Recent research has demonstrated the potential of utilizing mouse-tracking as a viable alternative method for examining attention-related attributes within the context of a multifaceted activity.

**Methods:**

In this study, a mouse-tracking technique was utilized to gather data from individuals who were involved in an online format of the Public Goods Game.

**Results:**

It was observed that participants exhibited distinct approaches to acquiring information while formulating decisions to propose high, moderate, or low offers. The mouse-tracking algorithm effectively distinguished between various types of offers made toward group funding, as evidenced by the measured distance of the cursor.

**Discussion:**

These findings suggest that mouse-tracking is a valuable tool for capturing decision-making processes and differentiating behavioral patterns in economic game contexts, offering insights into attention and choice mechanisms.

## Introduction

A public good refers to a type of good that offers advantages to all those involved, irrespective of their level of commitment toward its provision (Olson, 2012). The Public Goods Game (PGG), also known as the Voluntary Contribution Mechanism, as described by (Isaac and Walker 1988), is a commonly used model for studying public good challenges. The activity involves a collective of performers, wherein each individual is allocated a certain amount of resources and is faced with the decision of either retaining their allocation or contributing it to a communal fund designated for the group. In the context where all actors make contributions, the aggregate of their respective contributions is subjected to multiplication by a coefficient, typically leading to a sum greater than the initial contributions, followed by an equitable division among all participants. Therefore, the attainment of an optimal outcome for the group is facilitated through mutual cooperation, wherein the collective group balance is maximized by ensuring equal contributions from all players involved.

The cost-efficiency of a public product might give rise to conflicts between the benefits accrued collectively and those obtained individually. Certain individuals may have a preference for maximizing their personal earnings. This phenomenon occurs due to the fact that an individual's endowment diminishes in size when they choose to contribute it to the collective, as opposed to retaining it for personal benefit. Hence, it can be argued that adopting a rational approach entails maximizing personal benefits via abstaining from contributing to the collective fund, a behavior commonly referred to as free-riding. These behavioral patterns can also be shaped by cultural context. (Herrmann et al. 2008) demonstrated substantial cross-cultural variation in cooperative behaviors and antisocial punishment in Public Goods Games, including stark differences between Russia and Switzerland. This makes our choice of samples particularly relevant to examining whether behavioral dynamics in contribution also manifest in cognitive process measures like mouse tracking.

The hypothesis of complete free-riding in studies using the Public Goods Game (PGG) is frequently contradicted. In both one-shot Public Goods Games (PGGs) and recurrent PGGs with changing group composition after each round, it is commonly observed that contributions tend to average around 50% of the initial endowment (Walker and Halloran, 2004). However, in the subsequent scenario, contributions exhibit a gradual decrease, eventually reaching comparatively diminished levels in subsequent rounds.

This phenomenon can be elucidated by two primary categories of individual preferences (Ostrom, 2000; Fehr and Gintis, 2007; Ones and Putterman, 2007). Actors belonging to the first category are characterized by their rationality and self-oriented nature, displaying a lack of involvement in activities that benefit the broader community. The subsequent category of performers comprises conditional cooperators, who exhibit a tendency to contribute more when they anticipate higher contributions from others (Chaudhuri, 2011). These conditional cooperators adapt their expectations regarding the contributions of their peers based on their prior encounters with the average group contribution in preceding iterations (Fischbacher and Gächter, 2010). The degree to which individuals who exhibit conditional cooperation align their contributions with those of others exhibits variation on an individual basis. There exist individuals who exhibit imperfect conditional cooperation. In the context of repeated Public Goods Games (PGGs), it is common for individuals to engage in the observation of others' behaviors. When presented with the chance to react to the behavior of their counterparts, specifically in terms of rewarding or punishing, individuals tend to opt for punishment toward those that exhibit selfish behavior (Fehr et al., 2002). Nevertheless, the current availability of robust methods for analyzing the behavioral characteristics of players in Public Goods Games (PGG) is limited, hindering the ability to effectively detect their strategies on a trial basis.

Over the past 10 years, the utilization of mouse tracking has gained significant popularity as a methodological approach in various domains of psychology. This technique has been employed to investigate the intricate dynamics of cognitive processes, including but not limited to general decision making (Koop and Johnson, 2013) and social cognition (Freeman and Ambady, 2010; Freeman et al., 2011). The mouse tracking method is utilized to document hand movements by capturing the precise location of the mouse pointer on the screen. This enables the measurement of several parameters, such as the time of the movement leading up to the final decision, the average speed of the mouse movement, and the total distance covered by the mouse before the final decision is made. The assumption is made that the direction of movement, whether toward or away from options, signifies the relative attraction of these alternatives at a specific moment in the decision-making process. Therefore, this method has the potential to enhance neuroeconomics research by facilitating a more comprehensive analysis of participants' strategies throughout trial periods and providing additional insights into their motivations and preferences toward the available options.

Mouse-tracking provides a continuous, dynamic measure of the decision-making process, capturing how individuals waver between alternatives in real time. In neuroeconomics, which integrates neural and behavioral data to model how economic choices are made, such process-level data can reveal internal conflict and hesitation not apparent from final choices alone (Freeman et al., 2011). Unlike traditional choice paradigms that only capture the end decision, mouse trajectories can indicate underlying cognitive dynamics, such as indecision, approach-avoidance tendencies, and attraction toward competing alternatives. This makes mouse-tracking a powerful tool for studying strategic reasoning, particularly in social contexts like the Public Goods Game, where motivations such as reciprocity, trust, and fairness play central roles.

In this study, we have successfully included a mouse tracking technique into an online investigation of a Public Goods Game. The primary objective of this study is to ascertain whether the utilization of the mouse-tracking evaluation can yield further insights into the decision-making process of players on a trial-by-trial basis. Our hypothesis posits that there will be significant differences in mouse tracking metrics, such as speed, movement duration, and total distance, when participants make choices that contribute low, middle, or high amounts to the collective pool. We have selected two groups of participants from different cultural settings that we will treat as internal replication and initial evidence for generalizability.

## Methods

### Participants

A total of 116 participants were included in this study, comprising 50 individuals who spoke Russian (28 females) from Russia, and 66 individuals who spoke English (39 females) from Switzerland. Data collection took place between December 2020 and February 2021, with participants ranging in age from 18 to 30 years. The selection of Russian- and English-speaking participants aimed to provide a form of internal replication across culturally distinct populations. Prior work, notably (Herrmann et al. 2008), has shown systematic differences in cooperation and punishment behavior across cultures. By including these two groups, we sought to test whether mouse-tracking metrics would generalize across settings, or whether cultural norms would moderate strategic behavior and its cognitive correlates.

To further investigate heterogeneity in contribution behavior, we classified participants into strategy types following the typology developed by (Fischbacher et al. 2001) and (Fischbacher and Gächter 2010). This classification was based on participants' trial-by-trial responses in the “trials” condition, where they repeatedly made contribution decisions in the presence of simulated co-players. For each trial, we calculated the average contribution of the other players by computing the mean of three pre-defined simulated partner contributions (Player1, Player2, Player3).

Using each participant's full set of trials, we plotted their contribution as a function of the average contribution of others. Participants were classified according to the following criteria: “Free riders” consistently contributed near zero across all trials (all responses ≤ 0.5 tokens), “Altruists” consistently contributed near the maximum (all responses ≥5.5 tokens), “Conditional cooperators” exhibited a positive linear relationship between others' average contributions and their own, defined by a linear regression slope >0.1 (contributio*n* = **β*0*** +**β*1*** ·avg_others, where **β*1*** > 0.1), “Spiteful types” showed a negative linear relationship with slope <−0.1 (contributio*n* = **β*0*** +**β*1*** ·avg_others, where **β*1*** < 0.1), contributing less as others contributed more, “Hump-shaped contributors” exhibited a peaked response pattern, contributing most when others contributed a moderate amount, and less when others contributed very low or very high amounts (i.e., the maximum contribution occurred at an intermediate value of others' contributions and exceeded both endpoints), “Other” participants whose behavior did not fit any of the above patterns (e.g., inconsistent or random contributions). This classification allowed us to examine how strategy types differed in their use of decision time and mouse movement patterns, and also provided a behavioral explanation for inter-individual and cross-cultural variability.

To further examine the disparities in mouse-tracking variables, we conducted a thorough examination of the participants' responses. For this part we exclusively considered individuals who made contributions at all levels (high, middle, and low) to the collective pool for inclusion in the final analysis (please refer to the Statistical analysis section for further details). Consequently, this analysis incorporated data from a total of 30 participants who were proficient in Russian (17 females) and resided in Russia, as well as 29 respondents who were proficient in English (17 females) and resided in Switzerland. The data were obtained using the Gorilla.Sc Experiment Builder platform.

The study protocol was approved by the local university ethics committee: the HSE Committee on Interuniversity Surveys and Ethical Assessment of Empirical Research. All individuals participated in the study signed electronically the written informed consent form.

### Public Goods Game

In order to assess the inclination of individuals toward cooperative or egoistic free-riding behavior, we employed a modified version of the finitely repeated Public Goods Game (Sefton et al., 2007) including a group of four participants (one human participant and three computer models). The dataset obtained from (Sefton et al. 2007), graciously shared by the authors, was utilized to present our study participants with decision alternatives made by members of other, previously-tested groups (i.e., the three computer partners played according to the strategies observed in previous human players). Prior to the commencement of the game, participants were extended an invitation to partake in a Zoom session comprising multiple individuals. This obligatory Zoom meeting gave the perception that the individuals involved were engaging in live interactions with each other, as opposed to computer-generated simulations based on previously collected human behavior.

**Figure 1:**
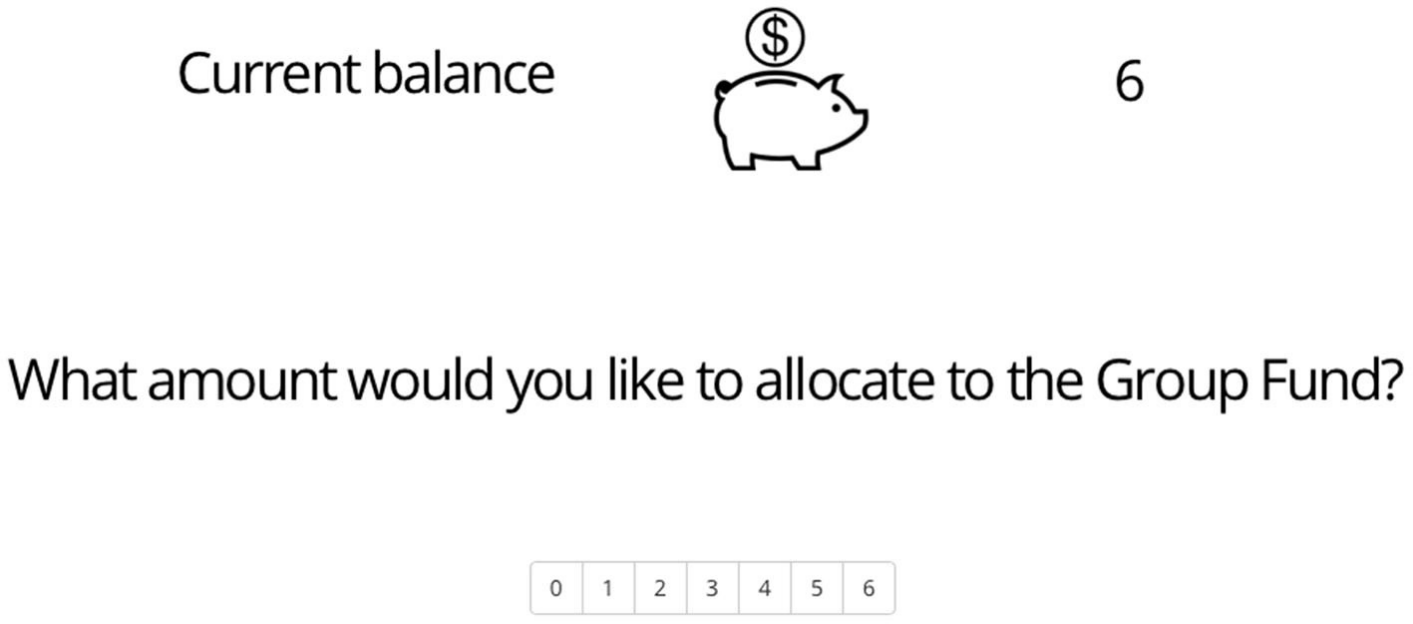
The layout of the response options in Public Goods Game.

At the commencement of each round, a total of 6 tokens were provided to the participant, who had the discretion to distribute these tokens between the Private Account and the Group Fund (see Figure 1 for the layout of response options). This process was repeated for a total of 10 rounds. The Group Fund was reconstituted in each round using the tokens that were contributed by the group members. Upon the conclusion of the round, the money in the Group Fund was equitably allocated among all members comprising the group. The conclusion of the round was marked by the allocation of tokens from the Group Fund.

In the punishment/reward phase, each participant was informed of the other three group members' contributions in the current round. Participants received an additional 6 tokens, which they could allocate to reward or punish one group member per trial. Allocating tokens for reward would increase that player's payoff by a fixed ratio, while allocating punishment would decrease the targeted player's payoff by the same amount. Any unused tokens were retained as personal earnings. Only one player could be targeted per round, and the decision was made after viewing contribution histories. The reward for the experiment was determined by analyzing the outcomes of the game. In both the Russian-speaking and English-speaking samples, each token that was collected on a Private Account was compensated with a payment of 10 kopecks or 10 Rappen, respectively. Each token disbursed from the Group Fund was remunerated at a value of 5 kopecks (in the Russian-speaking sample) or 5 Rappen (in the English-speaking sample).

### Mouse-tracking

The positioning of the mouse tracking zone occurred on the screen during the initial trial phase. Participants were provided with access to a range of accessible options to contribute to the group pool: they were given with a line of buttons numbered from 1 to 6, from which they could make their selection. The system enabled the identification and logging of the specific location on the screen where a participant's mouse cursor was positioned during the selection of the contribution amount. The mouse cursor started on each trial at a random point below the contribution number line. The data file produced by the Gorilla.Sc platform algorithm includes the temporal sequence of the participant's mouse position coordinates on the screen. Each position is accompanied by a corresponding timestamp, as well as additional information such as the x-coordinate, y-coordinate, width, and height of the participant's screen zone. In order to conduct a more comprehensive analysis, we utilized automatically generated normalized coordinates. These coordinates were expressed as a proportion of the Gorilla screen space. This approach enabled us to make meaningful comparisons of the coordinates between participants who used different screens or monitors. The mouse-tracking data were subsequently transformed from the Gorilla mouse-tracking data format to the format of the “mousetrap” R package (Wulff et al., 2021). This conversion was done in order to utilize the mousetrap functions for the computation of movement time, speed, and distance. The comprehensive R script containing the necessary details on the calculation of movement duration, speed, and distance can be accessed on the Open Science Framework website (https://osf.io/kgd92/?view_only=f196605985294b9c9eaa33254ce2d367).

### Statistical analysis

To organize the responses of the participants, we classified their results into three distinct categories based on the level of contribution they made to the group pool. These categories were defined as high contribution (5–6 tokens), middle contribution (3–4 tokens), and low contribution (0–1 and 2 tokens). Subsequently, the responses within each group were computed as an average and subjected to analysis using Repeated-Measures ANOVA. The ANOVA had three levels of one factor and the Bonferroni correction was applied to account for multiple comparisons for each mouse-tracking variable of interest, namely movement length, speed, and total distance. The chosen level of significance was established at α ≤ 0.05. To check the correlation between metrics, we have calculated Pearson coefficient. The data were analyzed using IBM SPSS 27.

After that, we have implemented an ordinal regression model with scikit-learn package in Python, using Offer as a dependent variable (encoded for the data analysis as 1—Low, 2—Middle, 3—High) and Duration, Speed and Distance as independent variables.

## Results

### English-speaking participants

Using trial-by-trial data to classify each participant into a strategy type using the (Fischbacher et al. 2001) typology, we identified 28 “spiteful”, 20 “conditional cooperators”, 12 “hump-shaped contributors”, 1 altruistic and 5 subjects considered unclassifiable.

To examine whether mouse-tracking metrics varied as a function of participants' contribution strategies, we conducted a one-way ANOVA on movement duration, average speed, and distance, excluding the Altruist group due to insufficient sample size (*n* = 1). The results revealed a statistically significant effect of strategy type on movement duration, *F*_(3,373)_ = 4.08, *p* = 0.0069, and average speed, *F*_(3,373)_ = 4.33, *p* = 0.0049. However, the effect on distance was not statistically significant, *F*_(3,373)_ = 2.01, *p* = 0.111. The descriptive statistics can be found in Table 1.

**Table 1:** Descriptive statistics for both groups.

**Variable**	**High offers (EN), ms**	**Middle offers (EN), ms**	**Low offers (EN), ms**	**High offers (RUS), ms**	**Middle offers (RUS), ms**	**Low offers (RUS), ms**
Movement duration (Mean)	2,243.6443	3,699.8298	3,090.6144	3,941.0302	7,108.5387	8,088.4416
Movement duration (SD)	1,414.8995	2,948.6034	1,795.5169	3,117.6599	7,303.8992	7,218.6662
Distance (Mean)	211.2595	189.9239	196.1028	221.4539	199.6264	199.6339
Distance (SD)	24.9022	27.5355	25.0128	37.8293	37.4121	36.4578
Average speed (Mean)	0.1494	0.1109	0.1207	0.1226	0.0878	0.0870
Average speed (SD)	0.0730	0.0616	0.0824	0.0850	0.0607	0.0629

Bonferroni-corrected *post hoc* comparisons indicated that “conditional cooperators” had significantly longer movement durations than “hump-shaped” participants (*p* = 0.028). No other pairwise differences reached statistical significance after correction.

### English-speaking participants (all types of offers presented)

For this analysis we exclusively considered individuals who made contributions at all levels (high, middle, and low) to the collective pool for inclusion in the final analysis, which resulted in 29 respondents who were proficient in English (17 females) and resided in Switzerland.

#### Movement duration

Repeated-Measures ANOVA showed that the movement duration between high, low and middle offers significantly differed [**F**_(1,821, 50, 987)_ = 4,007, ***p*** = 0.028, partial eta-square = 0.125], but this difference was not significant after Bonferroni correction for multiple comparisons.

#### Distance

Repeated-Measures ANOVA showed that the tracked mouse distance between high, low and middle offers significantly differed [**F**_(2,56)_ = 27,409, ***p*** < 0.001, partial eta-square = 0.495]. After correction for multiple comparisons there was a significant difference in distance between high and low offers (***p*** < 0.001), high and middle offers (***p*** < 0.001), but not low and middle offers (***p*** = 0.312).

#### Average speed

Repeated-Measures ANOVA showed that the average speed between high, low and middle offers significantly differed [**F**_(1,862,52,141)_ = 3,817, ***p*** = 0.031, partial eta-square = 0.120]. After correction for multiple comparisons there was a significant difference in distance between high and middle offers (***p*** = 0.017), but not between high and low offers and middle and low offers (***p*** > 0.05).

### Russian-speaking participants

Using trial-by-trial data to classify each participant into a strategy type using the (Fischbacher et al. 2001) typology, we identified 12 “spiteful”, 23 “conditional cooperators”, 9 “hump-shaped contributors”, 2 altruistic and 4 subjects considered unclassifiable.

To test whether decision dynamics varied by behavioral strategy type in the Russian-speaking sample, we conducted a one-way ANOVA on movement duration, average speed, and movement distance across four contribution types (excluding the Altruist group due to small sample size, *n* = 2). The analysis revealed a statistically significant difference in mouse movement distance, *F*_(3,N)_ = 2.67, *p* = 0.047, suggesting some variability in how far participants moved their cursor depending on strategy type. However, no significant effects were observed for movement duration, *F*_(3,N)_ = 1.48, *p* = 0.221, or average speed, *F*_(3,N)_ = 0.46, *p* = 0.711.

Bonferroni-corrected *post hoc* comparisons did not identify any statistically significant pairwise differences between strategy groups on any of the three mouse-tracking metrics, indicating that although overall differences in distance were significant, they did not localize to specific group comparisons after correction.

#### Russian-speaking participants (all types of offers presented)

For this analysis we exclusively considered individuals who made contributions at all levels (high, middle, and low) to the collective pool for inclusion in the final analysis, which resulted in 30 respondents who were proficient in Russian (17 females) and resided in Russia.

#### Movement duration

Repeated-Measures ANOVA showed that the movement duration between high, low and middle offers significantly differed [***F***_(2,58)_ = 4, 618, ***p*** = 0.014, partial eta-square = 0.137]. After correction for multiple comparisons there was a significant difference in distance between high and middle offers (***p*** = 0.006) and high and low offers (***p*** = 0.040) but not between middle and low offers (***p*** > 0.05).

#### Distance

Repeated-Measures ANOVA showed that the tracked mouse distance between high, low and middle offers significantly differed [***F***_(1,879,54,489)_ = 10,856, ***p*** < 0.001, partial eta-square = 0.227]. After correction for multiple comparisons there was a significant difference in distance between high and middle offers (***p*** = 0.003) and high and low offers (***p*** < 0.001) but not between middle and low offers (***p*** > 0.05).

#### Average speed

Repeated-Measures ANOVA showed that the average speed between high, low and middle offers significantly differed [**F**_(1,746,50,643)_ = 3,508, ***p*** = 0.043, partial eta-square = 0.108]. However, this difference was not significant after Bonferroni correction for multiple comparisons.

The effect sizes observed (partial η^2^ ranging from 0.108 to 0.495) indicate medium to large effects, particularly for distance. For instance, in both groups, high offers were associated with up to 15–20% greater mouse movement distance than low offers, suggesting increased cognitive engagement and possibly greater social deliberation.

## Correlation between metrics

### English-speaking participants

When checked for high offers, Pearson correlation coefficient was significant for duration and average speed (***r*** = −0.727, *p* < 0.001) and distance and speed (***r*** = 0.331, ***p*** = 0.034).

When checked for middle offers, Pearson correlation coefficient was significant for duration and average speed (***r*** = −0.640, ***p*** < 0.001), but not significant for other pairwise comparisons.

When checked for low offers, Pearson correlation coefficient was significant for duration and average speed (***r*** = −0.756, ***p*** < 0.001) and distance and speed (***r*** = 0.298, ***p*** = 0.027).

When responses to all types of offers (high, middle and low) were aggregated, Pearson correlation coefficient was significant for duration and average speed (***r*** = −0.675, ***p*** < 0.001) and distance and speed (***r*** = 0.242, ***p*** = 0.03).

### Russian-speaking participants

When checked for high offers, Pearson correlation coefficient was significant for duration and average speed (***r*** = –0.355, ***p*** = 0.021), but not significant for other pairwise comparisons.

When checked for middle offers, Pearson correlation coefficient was significant for duration and average speed (***r*** = −0.416, ***p*** = 0.004) and distance and speed (***r*** = 0.329, ***p*** = 0.024).

When checked for low offers, Pearson correlation coefficient was significant for distance and speed (***r*** = 0.520, ***p*** = 0.001), but not significant for other pairwise comparisons.

When responses to all types of offers (high, middle and low) were aggregated, Pearson correlation coefficient was significant for duration and average speed (***r*** = −0.409, ***p*** < 0.001) and distance and speed (***r*** = 0.294, ***p*** = 0.001).

## Ordinal regression

### English-speaking participants

The full results of the ordinal regression model can be found in Table 2. The log-likelihood of the model was found as −160.40, with AIC = 330.8 and BIC = 345.9.

**Table 2:** Ordinal regression model coefficients on English-speaking participants.

**Variable**	**Coefficient**	**Std. error**	***z*-value**	***P* > |*z*|**	**Conf. interval lower**	**Conf. interval upper**
Duration	−0.0001	9.95e−05	−1.258	0.209	−0.000	6.99e−05
Distance	0.0104	0.005	2.155	0.031	0.001	0.020
Speed	0.0146	2.846	0.005	0.996	−5.564	5.594
1/2	1.0773	0.987	1.092	0.275	−0.856	3.011
2/3	0.4786	0.119	4.10	0.000	0.245	0.712

The coefficient for duration was −0.0001, with a *p*-value of 0.209, indicating that it is not statistically significant. This suggests that changes in duration do not have a meaningful impact on the Offer. The coefficient for distance was 0.0104, with a *p-*value of 0.031, which was statistically significant, indicating that for each unit increase in distance, the odds of a higher offer increase, suggesting a positive relationship. The coefficient for Speed was 0.0146, with the *p* = 0.996, indicating that it was not statistically significant. This suggests that speed did not have a meaningful impact on the Offer.

We have also calculated the thresholds between the categories of Offer, with the first threshold (1/2) having a coefficient of 1.0773 (***p*** = 0.275) and the second threshold (2/3) having a coefficient of 0.4786 (***p*** = 0.000). The second threshold is statistically significant, indicating a meaningful difference between the second and third categories of Offer (middle and high offer, respectively).

### Russian-speaking participants

The full results of the ordinal regression model can be found in Table 3.

**Table 3:** Ordinal regression model coefficients on Russian-speaking participants.

**Variable**	**Coefficient**	**Std. error**	***z*-value**	***P* > |*z*|**	**Conf. interval lower**	**Conf. interval upper**
Duration	−0.3670	0.1865	−1.9681	0.0491	−0.7326	−0.002
Distance	0.2671	0.18	1.4842	0.1378	−0.0856	0.6198
Speed	0.2321	0.20	1.1668	0.2433	−0.1578	0.6221
1/2	−0.9457	0.2031	−4.6556	0.00000323009030713851	−1.3439	−0.5476
2/3	0.5211	0.1294	4.02733	0.0000564150118917629	0.2675	0.7747

The coefficient for the Duration was marginally significant (***p*** = 0.049), while the coefficients for Distance and Speed were not statistically significant.

We have also calculated the thresholds between the categories of Offer, which remained significant between categories 1 and 2 (Low and Middle, *p* < 0.001) and between 2 and 3 (Middle and High, *p* < 0.001).

## Between-group comparison

To examine whether mouse-tracking metrics differed as a function of both contribution strategy and participant nationality, we conducted a series of two-way ANOVAs on movement duration, average speed, and distance, including country group (Swiss vs. Russian) and strategy type (excluding altruists) as between-subjects factors. A total of nine tests were conducted (three metrics × three effects), and *p-*values were adjusted using the Bonferroni correction for multiple comparisons.

The analysis revealed a significant main effect of country group on movement duration, *F*_(1,1264)_ = 31.43, *p* < 0.001, which remained statistically significant after Bonferroni correction (*p* < 0.001). This indicates that Swiss and Russian participants differed systematically in the time they took to make decisions. Although group effects on average speed [*F*_(1,1,264)_ = 6.31, uncorrected *p* = 0.012] and distance [*F*_(1,1,264)_ = 21.81, uncorrected *p* < 0.001] appeared statistically significant before correction, neither remained significant after Bonferroni adjustment (*p* = 1.000 and *p* = 0.557, respectively).

No main effects of strategy type were significant for movement duration or average speed (*all adjusted p* > 0.10), and although interaction effects approached significance for movement duration [*F*_(3,1,264)_ = 2.47, *p* = 0.062] and average speed [*F*_(3,1,264)_ = 2.49, *p* = 0.060], these too did not survive Bonferroni correction (*p* = 1.000 and *p* = 0.211, respectively). Thus, only movement duration showed a robust difference between Swiss and Russian participants, regardless of contribution strategy.

## Discussion

In this study, we conducted an examination into the potential application of mouse-tracking technology in the study of economic decision-making, specifically in the context of a Public Goods Game (PGG). We found that strategy type has significant effect on movement duration in PGG and average speed detected by mouse-tracking in English-speaking participants (Swiss group), but not in Russian-speaking participants (Russian group). We also identified that in Swiss participants “conditional cooperators” had significantly longer movement durations than “hump-shaped” participants, suggesting higher cognitive effort or deliberation in their decision-making process, while in Russian participants mouse-tracking features did not significantly differ across subgroups with different strategy types.

Our study also revealed that among various geographically located groups, only one variable, namely the total distance covered by the mouse from the start of the trial to the decision point, exhibited a significant variation between high and low offers, as well as between high and middle offers. However, no significant difference in distance was observed between middle and low offers. In both groups, the distance covered for high offers was shown to be larger compared to that for low or moderate offers. Additionally, it was observed that among the English-speaking group (referred to as the Swiss group), the average speed of decision-making was revealed to be a significant predictor in differentiating between high and intermediate offers exclusively. With regard to our ordinal regression analysis, we also showed that distance has a positive predictive power for offers among English-speaking participants. Among Russian-speaking participants we found the reversed relationship between duration and offers, which was marginally significant.

Our results can be interpreted in light of the dual-process literature on prosocial behavior. Prior studies have shown that deliberation can either increase or decrease cooperation depending on context (Piovesan and Wengström, 2009; Rand et al., 2012). The longer trajectories and greater movement distances we observed in high contributors suggest that deliberation in this case facilitated socially optimal behavior, likely driven by the internal weighing of cooperative norms and personal costs.

Traditional behavioral metrics such as response time or frequency of cooperation provide outcome-level data. Mouse-tracking adds granularity by exposing sub-second fluctuations in preference that precede final decisions. This is particularly useful in decomposing strategic types (e.g., conditional cooperators vs. altruists) or identifying within-subject variability in repeated trials. Both eye-tracking and mouse-tracking measure dynamic attentional and cognitive processes, but they capture different aspects. Eye-tracking reflects visual attention and salience, while mouse-tracking reflects action preparation and decision conflict (Koop and Johnson, 2013; Freeman and Ambady, 2010). In social dilemmas, eye-tracking can reveal focus on self vs. others' outcomes (Fiedler et al., 2013), while mouse-tracking captures hesitation and strategic shifts. Combined, these methods offer a richer picture of decision-making. Neuroimaging studies have shown that cooperative decisions engage regions like the dorsolateral prefrontal cortex (for control) and ventromedial prefrontal cortex (for valuation). Mouse-tracking may serve as a low-cost, behaviorally anchored proxy for such neural dynamics by identifying when decisions are more effortful or automatic. Future studies could integrate EEG or fMRI with mouse-tracking to study the temporal and spatial unfolding of prosocial decisions. Prior studies in public goods research focus on aggregate contributions, conditional cooperation (Fischbacher et al., 2001), and punishment/reward effects. Our results contribute process-level data to this literature, showing that higher contributions are associated with more extensive cognitive-motor engagement as reflected in longer mouse trajectories, supporting theories of effortful deliberation in prosocial behavior.

Prior research in this particular domain has not thoroughly investigated the potential use of eye-tracking or mouse-tracking as a means to gather information about individuals' attention and gain insights into their strategies in economics games. This is despite the existence of specialized tools designed for the collection of ecologically valid data (Lejarraga et al., 2017). The studies conducted by (Kurihara and Funaki 2017) and (Peshkovskaya et al. 2017) demonstrated the efficacy of utilizing eye movements as a means to accurately anticipate individuals' actions in the context of preserving public goods. Nevertheless, significant research in this particular area can provide insights into the fundamental connection between sociocognitive elements, eye-movement patterns, and behaviors characterized by either selfishness or cooperation. (Fiedler et al. 2013) conducted a study that demonstrated a correlation between variations in social value orientation (SVO) and corresponding differences in information search patterns, as measured by eye-tracking technology. Specifically, the researchers found that decision time, number of fixations, proportion of inspected information, and number of transitions from and toward others' payoffs exhibited a gradual increase as the absolute deviation from a purely selfish orientation in SVO increased. Hence, eye-tracking offers direct evidence of visual attention, which in Public Goods Games can be used to infer norm compliance, fairness considerations, or strategic anticipation (Fiedler et al., 2013; Peshkovskaya et al., 2017). For instance, increased gaze to others' payoffs may predict conditional cooperation. Therefore, combining eye-tracking with mouse-tracking and neuroimaging can validate theoretical models of social preference by triangulating internal states across methods. Our study complements this by showing that greater cooperative intent is linked to more elaborate motor trajectories—interpretable as stronger internal deliberation or conflict resolution.

The results of our study indicate that there is a positive variation in information search and processing when individuals are more motivated to make cooperative decisions. This finding aligns with our observations, which showed that subjects who made higher offers toward the group pool shown a greater degree of distance compared to those who made middle or low offers. These findings also align with previous work by (Herrmann et al. 2008), which highlighted cultural divergences in cooperative tendencies and punitive norms. Our results suggest that these differences might not only be expressed in the outcome behavior but also in the cognitive effort and strategic consideration that precedes decisions, as captured by mouse-tracking metrics.

From a behavioral economics perspective, our study offers insight into the micro-processes underlying bounded rationality and conditional cooperation. From the standpoint of cognitive psychology, the observed mouse dynamics mirror theories of controlled vs. automatic processing, where effortful, strategic behaviors (like cooperation) manifest in more complex motor patterns (e.g., longer distances). This intersection offers a rich avenue for interdisciplinary inquiry.

Possible limitations of this study include possible skew toward longer distances for the high contribution options. Future studies might focus on further testing a mouse-tracking approach with different layouts of the response options on larger samples.

## Conclusion

Our research demonstrated the considerable potential of mouse-tracking as a viable approach for acquiring further understanding of participants' strategies in Public Goods Game (PGG). Specifically, our study revealed that distance was a significant factor in distinguishing the subjects' decision-making about high vs. intermediate or high vs. low contributions to the group fund. This finding was consistent across two separate groups, indicating its robustness across geographical and cultural settings. When strategy type was taken into account, it had a significant effect on movement duration in PGG and average speed detected by mouse-tracking in English-speaking participants (Swiss group), but not in Russian-speaking participants (Russian group). Specifically, “conditional cooperators” had significantly longer movement durations than “hump-shaped” contributors.

## Data Availability

The datasets presented in this study can be found in online repositories. The names of the repository/repositories and accession number(s) can be found below: https://osf.io/u2qmj/files/osfstorage?view_only=3b40e9cd341e4c26b7c685588c03e353.
