# Correction: Mouse-tracking as a tool for investigating strategic behavior in Public Goods Game: an experimental pilot study

**DOI:** 10.3389/fpsyg.2026.1807328

**Published:** 2026-02-20

**Authors:** Asma Benachour, Vladimir Medvedev, Oksana Zinchenko

**Affiliations:** 1 Laboratory of Brain-Computer Interfaces, Skolkovo Institute of Science and Technology, Moscow, Russia; 2 Centre for Neurocognitive Research, Moscow State University of Psychology & Education (MSUPE), Moscow, Russia; 3 Centre for Cognition and Decision Making, Institute for Cognitive Neuroscience, HSE University, Moscow, Russia

**Keywords:** mouse-tracking, Public Goods Game, decision-making, cooperation, strategic behavior

In the published article, affiliation Centre for Cognition and Decision Making, Institute for Cognitive Neuroscience, HSE University, Moscow, Russia was erroneously given as Centre for Cognition and Decision-Making, Institute for Cognitive Neuroscience, Moscow, Russia.

The original version of this article has been updated.

